# The Impact of Infliximab on Hyperinflammation State in Hospitalized COVID-19 Patients: A Retrospective Study

**DOI:** 10.3390/medicina60101670

**Published:** 2024-10-11

**Authors:** Yasmine M. Saied, Ahmed Essam Abou Warda, Rasha Mahmoud Allam, Wajid Syed, Mahmood Basil A. Al-Rawi, Ayesha Iqbal, Marwa O. Elgendy, Ramy M. El-Sabaa, Ahmed Hassan

**Affiliations:** 1 Microbiology and Immunology Postgraduate Program, Faculty of Pharmacy, Cairo University, Cairo 11562, Egypt; 2 Clinical Pharmacy Department, Faculty of Pharmacy, October 6 University, Giza 12585, Egypt; ahmed.essam@o6u.edu.eg; 3 Cancer Epidemiology and Biostatistics, National Cancer Institute, Cairo University, Cairo 11796, Egypt; allam.rasha@yahoo.com; 4 Department of Clinical Pharmacy, College of Pharmacy, King Saud University, Riyadh 11451, Saudi Arabia; wali@ksu.edu.sa; 5 Department of Optometry, College of Applied Medical Sciences, King Saud University, Riyadh 11451, Saudi Arabia; basilalravi@gmail.com; 6 Department of Pharmacy Practice and Policy, University Park Campus, University of Nottingham, Nottingham NG7 2QL, UK; ayesha.iqbal@nottingham.ac.uk; 7 Office of Lifelong Learning and the Physician Learning Program, Faculty of Medicine and Dentistry, University of Alberta, Edmonton, AB T6G1C9, Canada; 8 Department of Clinical Pharmacy, Beni-Suef University Hospitals, Beni-Suef University, Beni-Suef 62521, Egypt; marwa.elgendy@nub.edu.eg; 9 Department of Clinical Pharmacy, Faculty of Pharmacy, Nahda University (NUB), Beni-Suef 62764, Egypt; 10 Clinical Pharmacy Department, Faculty of Pharmacy, Menoufia University, Menoufia 32511, Egypt; ramy.mohamed@phrm.menofia.edu.eg; 11 Clinical Pharmacy Department, Faculty of Pharmacy, University of Sadat City, Menoufia 32897, Egypt; ahmhassancp@gmail.com

**Keywords:** infliximab, standard of care, pro-inflammatory cytokines, COVID-19

## Abstract

*Background and Objectives:* Elevated levels of pro-inflammatory cytokines have been linked to increased mortality in COVID-19 patients. Infliximab, a tumor necrosis factor inhibitor, has been reported to improve outcomes in COVID-19 patients by targeting the hyperinflammatory response. Our objective was to evaluate the effectiveness of incorporating Infliximab into standard care guidelines for the management of COVID-19. *Materials and Methods*: A retrospective analysis was conducted on 111 participants who were moderate to severe COVID-19 patients admitted to the hospital. Among them, 74 individuals received solely standard treatment, while 37 received standard therapy plus Infliximab. The primary outcomes of the study centered around the changes in laboratory test parameters. The secondary clinical findings included clinical recovery defined as improvement in patient oxygenation, time till recovery, and assessing necessity for ICU admission, and mortality rates. *Results:* There was no statistical difference observed in the inflammatory markers including, LDH, Ferritin, CRP, neutrophil to lymphocyte ratio (NLR), and P/F ratio between both groups and in the clinical outcomes including clinical recovery (*p* = 1.0), time to improvement (*p* = 0.436), and mortality rate (*p* = 0.601). However, there was a significant increase in secondary infection (45.9%, 20.3%; *p* = 0.005), and in liver enzymes, ALT (79.5, 50.0 IU/L; *p* = 0.02) and AST (57.5, 38.0 IU/L; *p* = 0.019) in the Infliximab group and the standard care group, respectively. *Conclusions:* Infliximab therapy did not demonstrate significant benefits compared to standard of care in moderate to severe hospitalized COVID-19 patients.

## 1. Introduction

The global outbreak of the coronavirus disease 2019 (COVID-19) resulted in a significant global health impact. As of the end of June 2024, the global count of recorded cases has surpassed 775 million, resulting in the number of fatalities exceeding seven million. In Egypt specifically, there have been over 500 thousand confirmed cases and a death toll of more than 24 thousand [1]. The COVID-19 pandemic caused by the SARS-CoV-2 virus, manifests with diversified symptoms varying in severity from mild to potentially fatal [2]. Various pharmaceutical strategies have been implemented in managing COVID-19, such as antiviral agents, antibodies, anti-inflammatory drugs, immunomodulatory therapies targeting specific pathways, and anticoagulants. The efficacy of these interventions varies across different patient demographics and disease phases and symptoms [3].

Current evidence indicates a variety of effective therapies for hospitalized COVID-19 patients, tailored according to the severity of their condition. Remdesivir is suggested for hospitalized patients with mild-to-moderate disease at high risk of progression to severe COVID-19, with its efficacy well supported by randomized controlled trials and real-world evidence. Dexamethasone is strongly recommended for patients with severe cases, particularly those requiring respiratory support. Additionally, for severe but non-critical COVID-19, IL-6 inhibitors (tocilizumab and sarilumab) and JAK inhibitors (baricitinib, preferred over tofacitinib) have shown a benefit when combined with corticosteroids. They are particularly advised for patients showing elevated inflammatory markers. While certain monoclonal antibodies were initially promising, their effectiveness depends on the circulating variants’ susceptibility [4].

A crucial discriminative characteristic among patients who successfully resolve the SARS-CoV-2 infection during its early phases and those who experience a severe disease state is the occurrence of cytokine storm syndrome. This phenomenon involves an inflammatory mechanism in which certain pro-inflammatory cytokines, including interleukin-6 (IL-6), interleukin-1 (IL-1), interferon (INF), IL-4, IL-7, IL-8, IL-9, IL-10, and tumor necrosis factor-α (TNF-α), play a crucial role [5]. The pro-inflammatory response plays a crucial role in the detrimental effects that viral infections have on the host and may be associated with more severe clinical consequences [6]. Tumor necrosis factor (TNF) plays a significant role in the commencement of the inflammatory cascade and is observed to be enhanced throughout the early stages of the disease process. Consequently, prompt management is of paramount importance. It regulates cell recruitment via the coordination of chemokines and adhesion molecules [7]. Moreover, it exacerbates lymphopenia through direct cytotoxic effects on T cells mediated by the TNF/TNFR1 signaling pathway [8]. Higher amounts of TNF, an important pro-inflammatory cytokine that promotes inflammation, have been associated with increased mortality in COVID-19 patients. The inhibition of TNF not only decreases the levels of biologically active TNF but also mitigates the production of other pro-inflammatory cytokines that contribute to the hyperinflammatory response observed in COVID-19 patients [7].

Several studies have reported a significant enhancement in the clinical condition of adult COVID-19 patients who received Infliximab [9,10,11], a chimeric monoclonal antibody commonly prescribed for the treatment of various autoimmune disorders such as rheumatoid arthritis, Crohn’s disease, and ulcerative colitis. Infliximab exhibits specific binding to and inhibition of TNF-α by overlapping the Infliximab Fab fragment with the TNFα-receptor interface. This interaction highlights the significant relevance of the E-F loop in the mechanism of action of this therapeutic antibody [12]. In comparison with corticosteroids, the use of TNF inhibitors possesses the potential to provide a more efficient approach in accelerating the recovery process of severe alveolar damage, which may arise as a consequence of influenza and coronavirus SARS infections [13].

Recent discussions have explored the potential preventive benefits of TNF inhibitors in managing severe COVID-19 cases [14]. Some studies have found no statistical difference between Infliximab and control groups regarding inflammatory markers such as C-reactive protein [15], time to recovery from COVID-19 pneumonia [16], and mortality rates [17].

Previous observational studies have presented evidence suggesting a possible link between the usage of anti-TNF medications and a lower probability of having negative events related to COVID-19 [5,18,19]. However, it is important to note that not all research groups have confirmed these findings [20]. This research assessed the outcomes of COVID-19 patients hospitalized and treated with both Infliximab and standard medication compared to those receiving only standard treatment.

## 2. Results

### 2.1. Baseline Characteristics

Both groups showed comparable statistical characteristics in terms of age (*p* = 0.606) and gender (*p* = 0.773). Also, the prevalence of pre-existing conditions such as hypertension, diabetes, chronic kidney disease, atrial fibrillation, and asthma was similar between the two groups. Notably, there were fewer patients with ischemic heart disease in the Infliximab combined with the standard care group (*n* = 3, 8.1%) compared to the standard care group (*n* = 18, 24.3%); (*p* = 0.040). Furthermore, a significant disparity was observed between the two groups concerning the severity of cases upon hospital admission, whereas the percentage of patients classified as severe (*p* = 0.002) was higher in the Infliximab group than in the standard care therapy group, Table 1.

The standard care therapy group exhibited superior oxygen saturation levels (*p* = 0.004), whereas the Infliximab group demonstrated a narrower respiratory rate (*p* = 0.026). IL-6 level at baseline was not statistically significant between both groups; (*p* = 0.515).

### 2.2. Medical Treatments

Lopinavir/ritonavir (Kaletra™, North Chicago, IL, USA) was used often in the Infliximab group; (*n* = 21, 56.8%) (*p* < 0.001), while hydroxychloroquine was used comparably in both groups (*p* = 0.575) (Table 1).

### 2.3. Associated Comorbidities

Associated comorbidities, including hypothyroidism, ulcerative colitis, cholecystectomy, hysterectomy, benign prostatic hyperplasia, previous percutaneous coronary intervention, valve disease, rheumatoid arthritis, osteoporosis, lymphoma, or hepatitis C virus, showed no difference in frequency between the two groups (*p* = 0.174) during the hospitalization course (Table 1).

### 2.4. Change in Monitoring Parameters within Each Treatment Protocol

No statistically significant differences were observed within each group before discharge regarding D-Dimer (ng/mL) and LDH (IU/L). However, ferritin levels (ng/mL) increased significantly within the group receiving Infliximab along with the standard care group (*p* = 0.025). Conversely, CRP levels (mg/dL) significantly decreased in both groups (*p* < 0.001).

TLC (cell count/μL) showed a statistically significant increase within each group (*p* = 0.01 for the Infliximab group, *p* < 0.001 for the standard care group), while the absolute lymphocyte count (%) statistically increased in the standard care group only (*p* = 0.005). Additionally, within the Infliximab plus standard care group, there was a statistically significant increase in the neutrophil-to-lymphocyte ratio (NLR) (*p* = 0.003) and improvement in the P/F ratio (*p* = 0.005) (Table 2).

### 2.5. Change in Monitoring Parameters between the Infliximab and Standard Care Groups

Before discharge, laboratory data including D-Dimer (ng/mL), LDH (IU/L), Ferritin (ng/mL), CRP (mg/dL), TLC (cell count/μL), absolute lymphocyte count (%), neutrophil to lymphocyte ratio (NLR), and P/F ratio were comparable between both groups. However, liver enzymes (ALT IU/L) showed a statistically significant difference (*p* = 0.02), as did AST (IU/L) (*p* = 0.019), with increased enzyme levels during hospitalization (Table 2).

### 2.6. Treatment Outcomes

There was no statistically significant difference in the number of patients who clinically improved (*p* = 1.0) or in the time to clinical improvement (*p* = 0.436) between the two groups. Similarly, the mortality rates did not differ significantly between the groups (*p* = 0.868).

While the need for invasive mechanical ventilation was low in both groups (*p* = 0.421), patients requiring ICU admission (86.5%; *p* = 0.001) and the use of non-invasive ventilation or high-flow oxygen (73.0%; *p* < 0.001) were notably higher in the Infliximab group. Also, the rate of secondary infection was higher in the infliximab group (*n* = 17, 45.9%; *p* = 0.005). Baseline serum creatinine (*p* = 0.656) and maximum temperature reading on the day of treatment initiation (*p* = 0.452) showed no differences between the groups (Table 3).

### 2.7. Survival Analysis

**Figure 1:**
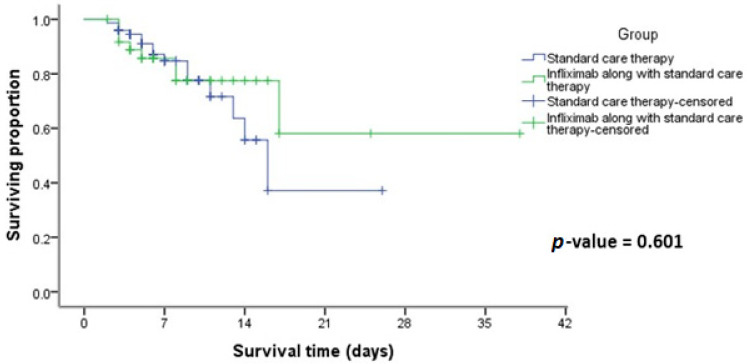
Kaplan–Meier survival curve of COVID-19 patients who received Infliximab along with standard care versus standard care alone.

A comprehensive observation of patients from the time of admission until their discharge and the total number of deaths were reported. Upon analysis, no statistical significance was found in survival rates between patients receiving Infliximab plus standard care and those receiving standard care alone (*p* = 0.601), as shown in Figure 1.

### 2.8. Risk Factors Associated with COVID-19 Severity through Logistic Regression Analysis

Logistic regression determined that COVID-19 severity upon admission was linked to respiratory rate (OR = 1.209, 95% CI 1.083–1.349, *p* = 0.001), and associated comorbidities (OR = 4.875, 95% CI 1.972–12.049, *p* = 0.001) (Table 4). However, after correcting for covariates with a *p*-value < 0.05 in the multiple logistic regression, only associated comorbidities remained significant (Adjusted OR = 4.875, 95% CI 1.972–12.049, *p* < 0.001) (Table 5).

## 3. Discussion

Therapeutic strategies to combat COVID-19 pneumonia are crucial, especially for communities that are disproportionately impacted by this pandemic across numerous ethnic and racial categories [21,22]. The emergence of acute respiratory distress can be attributed to the hyper-inflammatory state induced by the pathophysiology of COVID-19 [23,24]. Higher concentrations of the inflammatory mediator interleukin-6 (IL-6) have repeatedly been associated with serious development of this illness [25,26] and have been identified as a predictor of requiring ventilator aid [27].

TNF levels are elevated in COVID-19 patients, with higher baseline levels correlating with increased mortality risk. TNF inhibition represents a promising immunomodulatory approach for COVID-19 treatment [28,29], given its potential to mitigate inflammation, particularly the pro-inflammatory cytokines associated with adverse outcomes in COVID-19 patients [30]. However, Neurath’s research has raised doubts regarding the efficacy of TNF inhibitors in preventing severe outcomes in COVID-19 cases [14].

Our investigation revealed no significant change in clinical improvement or time to improvement between the Infliximab and standard care cohorts. In alignment, the ACTIV-1 IM trial, a large-scale, randomized, placebo-controlled clinical trial overseen by the National Institutes of Health, indicates that administering Infliximab or abatacept to hospitalized adults with COVID-19 did not significantly decrease time to recovery. However, it notably improved clinical status and reduced mortality rates [16]. Also, Farrokhpour et al. found no significant difference in hospital stay length between Infliximab and control groups in severe COVID-19 survivors but noted a disparity in ICU admission duration [13]. Supporting our study, the SECURE-IBD study examined the effects of anti-TNF monotherapy administration compared to a placebo. The study found no significant correlation between the use of TNF inhibitors and clinical outcomes such as the need for ventilator support or death [31].

Dexamethasone was uniformly administered to all patients across both the Infliximab and the standard care groups in our study. This universal application of dexamethasone reflects its established role in COVID-19 treatment protocols, particularly for its efficacy in reducing inflammation and mortality in severe cases. The RECOVERY trial has substantiated the benefits of dexamethasone, particularly in patients requiring respiratory support, by significantly lowering 28-day mortality rates. Further analysis from the trial underscores its role in reducing the risk of invasive mechanical ventilation for those on oxygen and improving the odds of weaning off for those already on support [32].

Our laboratory data, encompassing D-Dimer, LDH, Ferritin, CRP, TLC, absolute lymphocyte count (%), NLR, and P/F ratio, exhibited comparable values between both groups. In agreement, the CATALYST trial confirmed that Infliximab did not demonstrate any evidence of reducing inflammation, as indicated by CRP concentration, in COVID-19 patients who were admitted to hospital [15]. Additionally, while rituximab demonstrated a tendency toward a higher incidence of severe COVID-19 symptoms compared to Infliximab, both treatments showed similar rates [33].

In contrast, a recent study demonstrated that Tocilizumab/Infliximab treatment alongside standard care significantly reduced certain inflammatory markers, including CRP, LDH, absolute lymphocyte count, and NLR compared to Tocilizumab plus standard care in moderate/severe hospitalized COVID-19 patients. However, our results align with theirs regarding elevated liver enzymes ALT and AST [10]. In addition, Stallmach et al. reported that patients who received Infliximab, as opposed to supportive care, demonstrated an immediate decrease in inflammatory markers such as IL-6, CRP, and LDH levels, as well as notable clinical improvement [34].

Regarding our laboratory findings from baseline to post-treatment, TLC showed a statistically significant increase for the Infliximab group. In addition, there was a significant increase in NLR and an improvement in the P/F ratio. Similarly, Hachem et al.’s study results were consistent with our results in demonstrating an immediate recovery of lymphopenia in patients who initially had low levels of lymphocytes. These patients exhibited a significant increase in lymphocyte and monocyte counts upon discharge compared to baseline. Furthermore, patients receiving Infliximab exhibited improved respiratory metrics such as SpO_2_/FiO_2_ ratios and reduced dependence on mechanical ventilation [35].

Our research revealed no statistical significance in survival rates between patients receiving Infliximab plus standard care and those receiving standard care alone. Similarly, Fisher et al. found no difference in mortality rates, with death occurring in 14% of patients in the Infliximab group compared to 15% in the usual care group [15]. Moreover, a meta-analysis showed no significant difference in mortality rates between the Infliximab and control groups. However, Significant disparities were observed between patient groups concerning the timing of hospital discharge [17]. On the contrary, another study demonstrated statistical significance between Infliximab and control groups in survival rates among patients with severe COVID-19 [13].

In evaluating the clinical severity outcomes of COVID-19, our study highlights critical variables that significantly influence these endpoints. The multiple logistic regression analysis pinpointed associated comorbidities as a key determinant of increased disease severity upon hospital admission, echoing findings from other studies that consider ulcerative colitis [36], percutaneous coronary intervention [37], and rheumatoid arthritis [38] with worsened COVID-19 trajectories. These variables collectively elucidate the multifaceted nature of COVID-19, where underlying complications converge to dictate clinical outcomes.

While TNF inhibitors are extensively used to treat inflammatory diseases, it is noteworthy that not all immune-mediated inflammatory conditions are responsive. Additionally, TNF itself may exert suppressive effects on specific proinflammatory factors related to COVID-19, such as the expression of type 1 interferon and the differentiation of Th17 cells [39]. The inhibition of these cross-regulatory effects may account for the negative outcomes observed in our study, or it may suggest that TNF does not play a pivotal role in driving certain inflammatory responses in COVID-19 patients requiring hospitalization.

Our study exhibits some limitations. The small sample size hindered a definitive assessment of clinical outcomes, necessitating further investigations to elucidate the optimal patient populations for intervention. Additionally, our findings may not extend to non-hospitalized patients. The significant presence of severe cases and low oxygen saturation in the Infliximab group may introduce confounding variables that obscure interpretations.

## 4. Materials and Methods

### 4.1. Study Design

**Figure 2:**
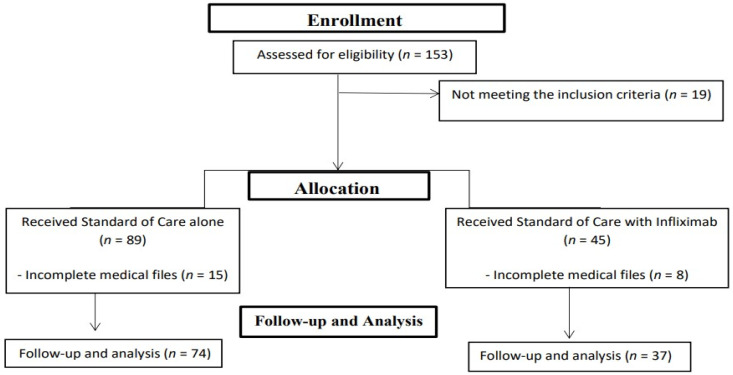
A flow chart of the study.

A retrospective analysis was undertaken on medical files of COVID-19 patients who were hospitalized at the Teachers’ Hospitals in Cairo, Egypt, between December 2020 and June 2022. The Research Ethics Board of the Faculty of Pharmacy, October 6 University granted approval for this study with the number PRC-Ph-2407001, ensuring compliance with the Declaration of Helsinki. The scope of our study encompassed two cohorts of hospitalized COVID-19 patients with moderate to severe symptoms. The control group (*n* = 74) received standard care therapy, whereas the intervention Group (*n* = 37) was given Infliximab along with standard of care, Figure 2.

### 4.2. Participants

Patients who were 18 years of age or older and met the following criteria were considered eligible: they were admitted to the hospital with a medical diagnosis strongly indicating SARS-CoV-2 pneumonia, which was confirmed by a chest X-ray or CT (computed tomography) scan and a positive RT-PCR (reverse transcription polymerase chain reaction) assay. Additionally, these patients encountered cytokine storm syndrome (CSS), as evidenced by elevated inflammatory markers. The markers used were a C-reactive protein (CRP) level of 100 mg/L or above, ferritin concentrations of 900 ng/mL or above, D-dimer levels larger than 750 ng/mL, lactate dehydrogenase (LDH) values surpassing 350 U/L, and interleukin-6 (IL-6) levels over 35 pg/mL. In addition, individuals who have an SpO_2_ level below 94% when breathing normal air at sea level, a (PaO_2_/FiO_2_) ratio below 300 mm Hg, a respiratory rate exceeding 30 breaths per minute, lung infiltrates greater than 50%, or demonstrate a deterioration in pulmonary consolidation characterized by an increase in the quantity and size of patches.

Exclusion criteria included recent use of immunosuppressive drugs, hypersensitivity to any TNFα inhibitors, prior COVID-19 vaccination, or a history of SARS-CoV-2 infection. Patient diagnoses that included a respiratory rate < 25 or a blood oxygen saturation > 95%, other active infectious diseases, and mental illness were also excluded.

All participants were provided with comprehensive information regarding the objectives and methods of the research. All patients were included in the study contacted to sign informed consent for using his/her hospital medical data to ensure the preservation of their confidentially. In cases where the patient’s capacity was determined to be lacking, in case of serious sickness or unavailability, the acquisition of informed consent was pursued by the patient’s legal representative. Patients who had provided representative consent were subsequently re-consented upon recovering their capacities.

### 4.3. Intervention

All patients received standard treatment, which involved an initial administration of 200 mg of remdesivir (Gilead Sciences, Foster City, CA, USA) following a daily maintenance dose of 100 mg. Also, 6 mg of dexamethasone (Pfizer Inc., New York, NY, USA) was administered once daily for at least seven days. Enoxaparin (Sanofi, Paris, France) was administered subcutaneously daily as a prophylactic measure when D-dimer levels were between 500 to 1000 ng/mL, and twice daily as a therapeutic intervention when levels exceeded 1000 ng/mL. Additional treatments included nightly quetiapine (AstraZeneca, Cambridge, UK) at 50 mg once daily and paracetamol (McNeil Consumer Healthcare, Fort Washington, PA, USA) every 6 h at 1 g doses. Besides the remdesivir, some patients who started treatments before admission were given either a daily dose of 200 mg of hydroxychloroquine (Sanofi, Paris, France) or a twice daily dose of 400/100 mg of lopinavir/ritonavir (AbbVie Inc., North Chicago, IL, USA). Patients allocated to the interventional group received a single intravenous dose of 5 mg/kg of Infliximab (Janssen, Horsham, PA, USA) in addition to their previous treatment.

### 4.4. Clinical Outcomes

All patients’ demographic information, medical history, laboratory data, medications, and complications were recorded from medical files by a clinical pharmacist. The primary outcome was the changes in laboratory tests throughout the hospital admission, including C-reactive protein (CRP), lactate dehydrogenase (LDH), D dimer, ferritin, Total lymphocyte count (TLC), Absolute Lymphocyte count, and Neutrophil to Lymphocyte Ratio (NLR).

Furthermore, secondary clinical outcomes were assessed based on clinical improvement or recovery, improving patient oxygenation, time to clinical improvement, ICU admissions, use of invasive or non-invasive ventilation, secondary infections, a threefold increase in liver enzymes above normal limits, and mortality rates. Additionally, safety outcomes consisted of daily examinations of hospitalized patients for potential complications, such as myocarditis, myocardial infarction, heart failure, pulmonary embolism, hypertension, and tachycardia. These adverse events were assessed by physicians in the COVID-19 department.

### 4.5. Statistical Analysis

#### 4.5.1. Sample Size Calculation

The analysis determined that 92 participants are required to achieve 80% power to detect an effect size f2 of 0.16 [13]. This analysis assumes a two-tailed hypothesis test with an alpha level of 0.05. The sample size calculation was performed by utilizing the G*Power (version 3.1.9.7) software developed by the University of Düsseldorf, Düsseldorf, Germany.

#### 4.5.2. Descriptive and Inferential Statistics

Continuous data presentation includes means and standard deviations. Categorical data are presented in number and percentage terms, while data normality was checked using Kolmogorov–Smirnov and Shapiro–Wilk tests. The Student’s *t*-test facilitated comparisons of means between two groups featuring numerical variables. Within each treatment group, a paired *t*-test was applied to evaluate disparities in clinical parameters before (baseline values) and after drug administration (post-treatment values). The Wilcoxon signed-rank and Mann–Whitney U tests were employed when analyzing paired and unpaired numerical variables over time of non-normally distributed data.

Also, the Chi-square test was applied to evaluate associations between categorical variables. Fisher’s exact test was used to analyze categorical data in small sample sets, and logistic regression was employed to identify clinical predictors of outcomes, with significance ascribed to variables achieving a *p*-value of 0.05 or lower.

The Kaplan–Meier approach and log-rank tests were employed to evaluate the time between admission and final release concerning mortality outcomes. All *p*-values reported were two-tailed, with a significance threshold set at less than 0.05. The data were analyzed using version 22.0 of the Statistical Package for the Social Sciences (SPSS) from Chicago, IL, USA, where the results were graphically or tabularly reported.

## 5. Conclusions

Our study revealed no significant benefits of Infliximab over standard care in hospitalized COVID-19 patients concerning clinical improvements, inflammation marker levels, and survival rates. Future research should focus on more targeted trials that include homogenous patient groups to ascertain the specific patient profiles that might benefit from TNF inhibition in COVID-19. Moreover, longitudinal studies could provide insights into the long-term effects of Infliximab treatment on COVID-19 recovery trajectories and help clarify its role in managing the disease.

## Figures and Tables

**Table 1:** Baseline characteristics in the studied groups.

Variables	SCT (*n* = 74)	Infliximab with SCT (*n* = 37)	*p*-Value
***Baseline characteristics***			
Age (years) **	61.5 (25.0–91.0)	63.0 (30.0–93.0)	0.606
Gender			
Male	50 (67.6%)	26 (70.3%)	0.773
Female	24 (32.4%)	11 (29.7%)	
Severity at Admission			
Moderate	34 (45.9%)	6 (16.2%)	0.002
Severe	40 (54.1%)	31 (83.8%)	
***Pre-existing Conditions***			
Hypertension	36 (48.6%)	18 (48.6%)	1
Heart Failure	2 (2.7%)	0 (0)	*
Ischemic Heart Disease	18 (24.3%)	3 (8.1%)	0.04
Atrial Fibrillation	6 (8.1%)	0 (0)	0.176
Diabetes Mellitus	36 (48.6%)	13 (35.1%)	0.176
Chronic Kidney Disease	3 (4.1%)	2 (5.4%)	1
Chronic Liver Disease	2 (2.7%)	0 (0)	*
Asthma	5 (6.8%)	1 (2.7%)	0.662
COPD	2 (2.7%)	1 (2.7%)	*
Associated Comorbidities	28 (37.8%)	19 (51.4%)	0.174
***Medications***			
Remdesivir	74 (100%)	37 (100%)	1
Dexamethasone	74 (100%)	37 (100%)	1
Lopinavir/Ritonavir (*n* = 31)	10 (13.5%)	21 (56.8%)	<0.001
Hydroxychloroquine (*n* = 15)	9 (12.3%)	6 (16.2%)	0.575
***Diagnostic parameters***			
IL-6 (pg/mL) **	66.5 (4.31–355.5)	77.0 (6.64–245.0)	0.515
Oxygen Saturation **	92.0 (50.0–97.0)	85.0 (40.0–97.0)	0.004
Respiratory Rate (breaths/minute) **	24.5 (13.0–37.0)	27.0 (18.0–35.0)	0.026

* The *p*-value cannot be calculated because of the small number within strata. ** Data are represented as the median (range). SCT: standard care therapy.

**Table 2:** Comparison of the studied groups for the change in the laboratory parameters within each treatment (*p*-value time effect) and between treatments (*p*-value group effect).

Variables	SCT (*n* = 74) Median (Range)	Infliximab with SCT (*n* = 37) Median (Range)	*p*-Value (Group Effect)
***CRP (mg/dL)***			
Baseline laboratory results	102.0 (3.5–312.0)	124.0 (6.6–390.0)	0.517
Laboratory results before discharge	22.0 (0.6–444.0)	11.5 (0.7–447.0)	0.704
*p*-value (time effect)	<0.001	<0.001	
***D-Dimer (ng/mL)***			
Baseline laboratory results	0.4 (0.1–87.0)	0.6 (0.1–6.3)	0.718
Laboratory results before discharge	0.5 (0.1–38.6)	0.5 (0.1–19.0)	0.435
*p*-value (time effect)	0.938	0.769	
***Ferritin (ng/mL)***			
Baseline laboratory results	611.0 (26.0–4176.0)	506.0 (56.0–2564.0)	0.545
Laboratory results before discharge	559.0 (44.0–2918.0)	767.0 (72.0–2946.0)	0.427
*p*-value (time effect)	0.38	0.025	
***LDH (IU/L)***			
Baseline laboratory results	325.0 (137.0–1288.0)	373.0 (173.0–1434.0)	0.503
Laboratory results before discharge	301.0 (155.0–1247.0)	372.0 (148.0–1589.0)	0.06
*p*-value (time effect)	0.759	0.058	
***P/F ratio***			
Baseline laboratory results	162.0 (60.0–385.0)	135.0 (71.4–438.0)	0.656
Laboratory results before discharge	200.0 (68.0–333.0)	223.0 (110.0–329.0)	0.656
*p*-value (time effect)	0.066	0.005	
***TLC (cells count/μL)***			
Baseline laboratory results	6.9 (2.0–25.0)	8.0 (4.1–17.1)	0.105
Laboratory results before discharge	10.0 (3.4–35.8)	9.9 (2.6–22.5)	0.663
*p*-value (time effect)	<0.001	0.01	
***Absolute Lymphocyte count (%)***			
Baseline laboratory results	13.0 (2.0–34.0)	9.0 (5.0–31.0)	0.102
Laboratory results before discharge	9.5 (2.0–22.0)	6.0 (2.0–24.0)	0.187
*p*-value (time effect)	0.005	0.069	
***Neutrophil to Lymphocyte Ratio (NLR)***			
Baseline laboratory results	6.5 (0.9–41.0)	8.0 (0.5–22.0)	0.071
Laboratory results before discharge	9.3 (1.7–29.0)	11.8 (1.5–47.0)	0.115
*p*-value (time effect)	0.112	0.003	
***ALT (IU/L)***			
Baseline laboratory results	39.5 (14.0–382.0)	33.0 (5.9–151.0)	0.029
Laboratory results before discharge	50.0 (14.0–626.0)	79.5 (13.0–469.0)	0.02
*p*-value (time effect)	<0.001	<0.001	
***AST (IU/L)***			
Baseline laboratory results	42.0 (14.0–247.0)	38.0 (11.0–171.0)	0.358
Laboratory results before discharge	38.0 (14.0–262.0)	57.5 (17.0–920.0)	0.019
*p*-value (time effect)	0.99	0.01	

SCT: standard care therapy.

**Table 3:** Clinical outcomes in the studied groups.

Variables	SCT (*n* = 74)	Infliximab with SCT (*n* = 37)	*p*-Value
***Clinical outcomes***			
Clinical improvement	58 (78.4%)	29 (78.4%)	1
Time to clinical improvement (days) **	6.0 (4.0–25.0)	7.0 (4.0–22.0)	0.436
Need for ICU admission	40 (54.1%)	32 (86.5%)	0.001
Low supplemental oxygen	35 (47.3%)	7 (18.9%)	0.004
Non-invasive ventilation or high-flow oxygen	21 (28.4%)	27 (73.0%)	<0.001
Invasive mechanical ventilation	6 (8.1%)	1 (2.7%)	0.421
Temperature ^a^**	38.0 (36.8–40.0)	38.0 (37.0–40.0)	0.452
Serum creatinine (mg/dL) **	1.30 (0.70–7.10)	1.40 (0.80–15.0)	0.656
Death	15 (20.3%)	8 (21.6%)	0.868
***Complications***			
Secondary infection	15 (20.3%)	17 (45.9%)	0.005
Myocarditis	11 (14.9%)	4 (10.8%)	0.556
Myocardial infarction	1 (1.4%)	3 (8.1%)	*
Heart failure	2 (2.7%)	1 (2.7%)	*
Pulmonary embolism	3 (4.1%)	2 (5.4%)	1
Hypertension	1 (1.4%)	0 (0)	*
Tachycardia	2 (2.7%)	0 (0)	*

^a^ Maximum temperature on the day of treatment initiation °C. ** Data are represented as the median (range). SCT: standard care therapy. * The *p*-value cannot be calculated because of the small number within strata.

**Table 4:** Risk factors associated with COVID-19 severity during binary logistic regression analysis.

		Severity at Admission		COR (95% CI)	*p*-Value
Variables		Moderate (*n* = 40)	Severe (*n* = 71)		
***Baseline characteristics***					
Age (years)	Median (Range)	59.0 (25–91)	63.5 (30–93)	1.021 (0.988–1.054)	0.201
Gender	Male	28 (36.8%)	48 (63.2%)	Reference	0.794
	Female	12 (34.3%)	23 (65.7%)	1.118 (0.483–2.587)	
***Pre-existing Conditions***					
Hypertension	Yes	15 (27.8%)	39 (72.2%)	2.031 (0.919–4.488)	0.080
	No	25 (43.9%)	32 (56.1%)	Reference	
Heart Failure	Yes	0 (0)	2 (100%)	0.343 (0.016–7.327)	0.494
	No	40 (36.7%)	69 (63.3%)	Reference	
Ischemic Heart Disease	Yes	9 (42.9%)	12 (57.1%)	Reference	0.471
	No	31 (34.4%)	59 (65.6%)	1.427 (0.542–3.756)	
Atrial Fibrillation	Yes	2 (33.3%)	4 (66.7%)	1.134 (0.198–6.485)	0.887
	No	38 (36.2%)	67 (63.8%)	Reference	
Diabetes Mellitus	Yes	15 (30.6%)	34 (69.4%)	1.532 (0.694–3.380)	0.291
	No	25 (40.3%)	37 (59.7%)	Reference	
Chronic Kidney Disease	Yes	2 (40.0%)	3 (60.0%)	Reference	0.850
	No	38 (35.8%)	68 (64.2%)	1.193 (0.190–7.457)	
Chronic Liver Disease	Yes	0 (0)	2 (100.0%)	Reference	0.494
	No	40 (36.7%)	69 (63.3%)	0.343 (0.016–7.327)	
Asthma	Yes	2 (33.3%)	4 (66.7%)	1.134 (0.198–6.485)	0.887
	No	38 (36.2%)	67 (63.8%)	Reference	
COPD	Yes	0 (0)	3 (100%)	0.242 (0.012–4.798)	0.352
	No	40 (37.5%)	68 (63.0%)	Reference	
Respiratory Rate (breaths/minute)	Median (Range)	22.00 (13.00–31.00)	27.00 (16.00–37.00)	1.209 (1.083–1.349)	0.001
Max. Temp reading on treatment initiation day (°C)		38.00 (36.80–40.00)	38.00 (37.00–40.00)	1.197 (0.761–1.882)	0.436
Associated comorbidities	Yes	8 (17.0%)	39 (83.0%)	4.875 (1.972–12.049)	0.001
	No	32 (50.0%)	32 (50.0%)	Reference	

COR: crude odds ratio; CI: confidence interval.

**Table 5:** Multiple logistic regression analysis showing risk factors significantly associated with COVID-19 severity.

Variables	*p*-Value	AOR (95% C.I.)
Associated comorbidities (yes/no)	<0.001	4.875 (1.972–12.049)

AOR: adjusted odds ratio.

## Data Availability

The data supporting the findings of this study are contained within the article.
